# Detection of group A rotavirus strains circulating among children with acute diarrhea in Indonesia

**DOI:** 10.1186/s40064-016-1724-5

**Published:** 2016-01-29

**Authors:** Hera Nirwati, Tri Wibawa, Abu Tholib Aman, Abdul Wahab, Yati Soenarto

**Affiliations:** Department of Microbiology, Faculty of Medicine, Universitas Gadjah Mada, Yogyakarta, Indonesia; Department of Public Health, Faculty of Medicine, Universitas Gadjah Mada, Yogyakarta, Indonesia; Department of Paediatric, Faculty of Medicine, Universitas Gadjah Mada/Sardjito Hospital, Yogyakarta, Indonesia

**Keywords:** Rotavirus, G-type, P-type, Acute diarrhea

## Abstract

Rotavirus is the major cause of severe diarrhea in children under 5 years old in developed and developing countries. Since improvements in sanitation and hygiene have limited impact on reducing the incidence of rotavirus diarrhea, implementation of a vaccine will be a better solution. We conducted an observational study to determine the disease burden and to identify the genotype of circulating rotavirus in Indonesia. Hospitalized children due to acute diarrhea were enrolled from four teaching hospitals in Indonesia. Stool samples were collected based on WHO protocol and were tested for the presence of group A rotavirus using enzyme immunoassay. Then, rotavirus positive samples were genotyped using RT-PCR. Fisher’s Exact tests, Chi square tests and logistic regression were performed to determine differences across hospital and year in rotavirus prevalence and genotype distribution. There were 4235 samples from hospitalized children with diarrhea during 2006, 2009 and 2010. Among them, the rotavirus positive were 2220 samples (52.42 %) and incidence rates varied between hospitals. The G1P[8], G1P[6], and G2P[4] were recognized as the dominant genotypes circulating strains in Indonesia and the proportion of predominant strains changed by year. Our study showed the high incidence of rotavirus infection in Indonesia with G1P[8], G1P[6], and G2P[4] as the dominant strains circulating in Indonesia. These results reinforce the need for a continuing surveillance of rotavirus strain in Indonesia.

## Background

Rotavirus is a major cause of severe diarrhea in both developed and developing countries. Previous report estimates that more than 100 millions episodes of gastroenteritis requiring home care only. However, almost 25 million cases need clinical visit, 2 million cases need hospital admission, and 440,000 death cases in children under 5 years of age. By the age of 5, nearly every child experiences an episode of rotavirus gastroenteritis, 1 in 5 will visit a clinic, 1 in 65 will be hospitalized, and approximately 1 in 293 will die. The mortality rate in children under 5 years old is high which more than 80 percent occurs in Africa and South Asia (Parashar et al. 2003). In Indonesia, rotavirus was the major causative viral infection responsible for diarrheal diseases in children (37.5 %) followed by adenovirus (3.3 %) and Norwalk-like virus (17.6 %). Bacterial infection such as *Enterotoxigenic E*. *coli*, *Enterohemorrhagic E*. *coli* and *Clostridium difficile* had lower incidence (Putnam et al. 2007).

The incidence of rotavirus disease was observed to be similar in both industrialized and developing countries. Some previous studies reported that the rotavirus incidence in Indonesia varied between 38 and 61 % and the majority of infected children were under 2 years old (Putnam et al. 2007; Soenarto et al. 1981; Ofoyo et al. 2002; Soenarto et al. 2009; Radji et al. 2010). As vomiting was predominantly occurred in rotavirus diarrhea, the symptoms of rotavirus diarrhea can not be treated with rehydration therapy only, disease prevention by vaccination with long-term strategy is recommended (Parashar et al. 2009). In a projected birth cohort study of 4.2 million children with follow up for 5 years, routine rotavirus vaccination program could potentially avert 488,547 cases of diarrhea treated outpatient, 176,375 hospitalizations, and 8148 deaths (Wilopo et al. 2009).

Based on the facts above, a study of rotavirus epidemiology is needed to introduce appropriate rotavirus vaccine development and implementation. Due to the fact that the predominant serotypes variation which very unpredictable from year to year in particular location, vaccines must provide heterotypic protection (Gentsch et al. 2005). The decision to develop or to fund a rotavirus vaccine in Indonesia is hindered by the lack of awareness of the significant burden of rotavirus infection and the importance of rotavirus vaccine. Thus, performing a surveillance study of circulating rotaviruses and publishing its findings to community may raise awareness related to application of rotavirus vaccine.

This research is aimed to identify rotavirus strains causing diarrhea in children under 5 years old in Indonesia based on hospital surveillance and to describe the disease burden caused by rotavirus infection. The longitudinal nature of this research allows further observation on the changes of circulating rotavirus strains over time.

## Methods

As a member of Asian Rotavirus Surveillance Network (ARSN), a hospital surveillance was conducted by using a WHO standard protocol. We performed an observational and Hospital based study which conducted at Sardjito Hospital, Hasan Sadikin Hospital, Sanglah Hospital, and Mataram Hospital during 2006, 2009, and 2010. Hospitalized children were enrolled after informed and written consent obtained from the parents or guardians. A suspected case was defined as any case in a child under 5 years of age who was admitted to the hospital with acute diarrhea (≥3 stools looser than normal within 24 h) (Soenarto et al. 2009; World Health Organization 2002).

Approximately 3–5 ml stool sample was collected in the first 48 h of admission based on WHO protocol (World Health Organization 2002). The stool samples were transported to Microbiology Laboratory, Faculty of Medicine, Universitas Gadjah Mada. All stool samples were then aliquoted and stored at −20 °C until stool analysis.

All stool samples were tested for the presence of group A rotavirus using IDEIA™ Rotavirus (DakoCytomation) kit according to the manufacturer instructions. Stratified random sampling method based on sample collection timing and age was used to select rotavirus positive samples for genotyping study. Rotavirus RNA was extracted from rotavirus positive stool samples and analyzed to determine both the VP7 (G-type) and the VP4 (P-type) genotypes using method described by Das et al. (1994) or Gouvea et al. (1990) and Gentsch et al. (1992) or Simmond et al. (2008), respectively.

Fisher’s Exact tests, Chi square tests and logistic regression were performed to determine differences across hospital and year in rotavirus prevalence and genotype distribution.

The study protocol was approved by the Ethical Committee of the Faculty of Medicine, Universitas Gadjah Mada, Yogyakarta. The parents or guardians of each child signed a written informed concent prior to their child’s enrollment in the study.

## Results

In the four teaching hospitals, we found 4235 cases of diarrhea with 2537 (59.9 %) male and 1698 (40.1 %) female patients. Among 4235 cases, up to 3624 (86 %) cases were children under 24 months and 611 (14.45 %) cases were children aged 25–59 months. Rotavirus test using enzyme immunoassay showed that from all stool samples collected in the study, 2220 (52 %) samples were rotavirus positive. Out of them, a total of 517 samples (23 %) were randomly genotyped which consisted of 160, 183, and 174 samples collected in 2006, 2009, 2010, respectively from all hospitals (Table 1).

**Table 1:** Demographic characteristic of study subjects

Characteristic	Year					
	2006		2009		2010	
	RV positive	Number tested	RV positive	Number tested	RV positive	Number tested
Age (months)						
0–5	189	441	84	200	86	184
6–11	468	790	141	288	186	324
12–23	448	746	172	311	190	340
24–59	140	316	59	160	57	135
Sex						
Male	777	1384	278	565	316	588
Female	468	909	178	394	203	395
Hospitals						
Sardjito	116	353	45	149	32	99
Hasan Sadikin	87	184	131	290	132	248
Sanglah	355	605	104	239	144	306
Mataram	687	1151	176	281	211	330
	1245	2293	456	959	519	983

*RV* rotavirus

**Fig. 1:**
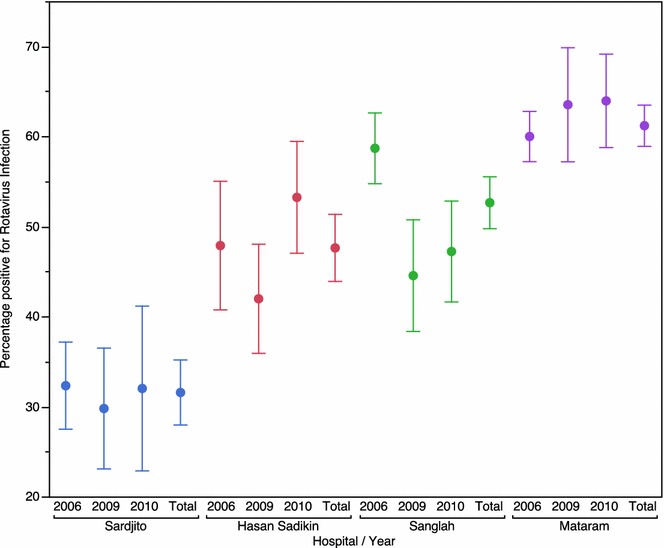
Percentage of positive identified rotavirus among young hospitalized children with diarrhea at four study hospitals. Data were shown as percentage ±95 % CI. Rotavirus detection was performed by using enzyme immunoassay methods. *Different collors* of chart are represent data obtained from different hospitals in 2006, 2009, 2010, and total percentage during three indicated years

Overall, the percentage of children who were rotavirus positive across the four hospitals varied from 30 to 64 %. Rotavirus prevalence among hospitalized children with diarrhea was significantly different between hospitals (p < 0.001; Fig. 1). This difference persisted after adjustment of age, gender, and year. During the study, Sardjito hospital had the lowest prevalence, whereas Mataram hospital had the highest prevalence.

**Fig. 2:**
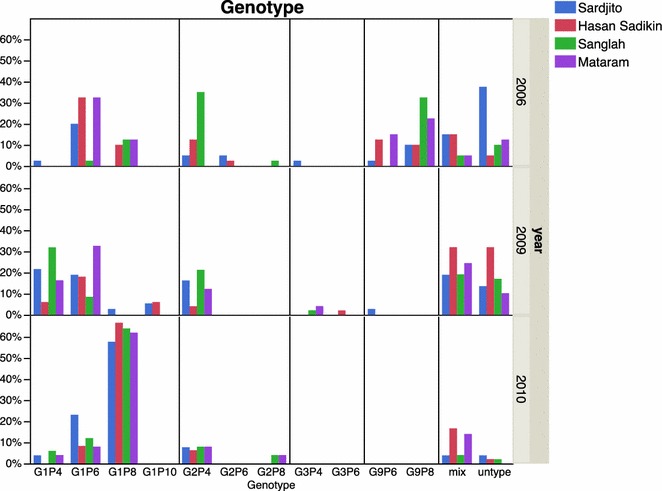
Distribution of rotavirus genotypes among young hospitalized children with diarrhea admitted to four study hospitals in 2006, 2009 and 2010. *Histogram* depicted in *different colors* represented four different hospitals. Genotyping was performed by using RT-PCR methods

Rotavirus genotype in three non-consecutive years showed different compositions. In 2006, the predominant genotypes were G1P[6] (21 %) and G9P[8] (21 %), followed by G9P[6] (13 %) and G2P[4] (10 %). In 2009, the predominant genotypes were G1P[6] (16 %), G1P[4] (16 %), G1P[8] (15 %), and G2P[4] (14 %). In 2010, G1P[8] was by far the most common by 64 % and smaller minority of infections made up of G1P[6] (11 %), G2P[4] (7 %), or G1P[4] (3 %) (Fig. 2).

## Discussion

We reported the high incidence of rotavirus infection in Indonesia that varied significantly between four teaching hospitals. Strikingly, compared to mostly previous hospital-based studies, the rotavirus prevalence rate in our study was higher.

We found that most of the strains circulating in Indonesia were similar to the strains circulating elsewhere in the world. Previous epidemiological studies estimated that four common G types (G1, G2, G3 and G4) in conjunction with P[8] or P[4] represented over 88 % of the strains analyzed worldwide. In addition, G9 associated with P[8] or P[6] were shown to have emerged as the fourth globally important G type strain (Santos and Hoshino 2005). A few studies have been conducted on rotavirus strains in Indonesia and reported the presence of G1, G2, G3, G4 and G9 in conjunction with P[4], P[6] and P[8] as the strains circulating in Indonesia (Putnam et al. 2007; Ofoyo et al. 2002; Soenarto et al. 2009; Radji et al. 2010; Bishop et al. 1989). Our study confirmed that the predominant rotavirus genotypes in Indonesia were varied by year and location, thus emphasizing the importance of continued surveillance on circulating genotypes.

Other Asian countries also reported the variation of rotavirus genotypes. Qazi et al. (2009) reported that infection causing genotypes of rotavirus in Pakistan were G9P[8], G1P[8], and G1P[4]. Cuong et al. (2009) reported that G3P[8] and G1P[8] became the predominant genotypes in Vietnam. Nyambat et al. (2009) reported that G1P[8] and G2P[4] were the predominant genotypes causing diarrhea in Cambodia. Moe et al. (2009) reported that G3P[8], G1P[8] and G1P[4] genotypes were the major cause of diarrhea in Myanmar. Finally, Khamrin et al. (2010) reported that the causes of diarrhea in Thailand were G1P[8], G2P[4], G9P[8], G3P[8], and G3P[10] genotypes.

Of our interest, we found uncommon rotavirus strains such as G1P[4], G1P[6], G1P[10], G2P[6], G2P[8], G2P[10], G3P[4], G3P[6], and G9P[6], as well as mixed infection and untypeable strains. These findings are in line with previous observation that in developing country, rotavirus strains circulating were more diverse compared to the one in developed country (Kawai et al. 2012; Glass et al. 2005; Glass et al. 2006; Esona et al. 2010). Moreover, mixed infections by several genotypes are mainly found in developing countries. In Japan and the United States, mixed infections are only found in less than 5 % of cases while in India and Brazil it reaches 10–25 % (Gentsch et al. 2005). Surveillance study in Argentina by Esteban (Esteban et al. 2010) reported that 18 % of infections were mixed. Collectively, mixed infection gives an opportunity for continuous evolution of the virus. Infection on one individual by two or more strains increases the likelihood of genetic exchange through strain re-assortment, making the emergence of a new strain more probable. Mixed infections were reported to occur more frequently in poor sanitation areas where gastrointestinal infections were more prevalent (Castello et al. 2004). Finally, our study confirmed that mixed infection in Indonesia was higher that could be caused by genetic exchange of rotavirus strain circulating.

It should be noted that our hospital based study was conducted only in four hospitals, therefore it might not necessary capture all strains circulating in Indonesia, or the overall incidence among children who were not hospitalized for diarrhea. Despite the limitations of this study, our findings support one of the key steps for policy making in deciding the importance of rotavirus vaccine implementation.

## Conclusion

Our study showed the high prevalence of rotavirus infection in Indonesia. Among known rotavirus strain circulating, G1P[8], G1P[6], and G2P[4] were identified as the dominant strains circulating in Indonesia. Because of genotypic changes by year, our study reinforces the need for a continuing surveillance of rotavirus strain circulating. In the near future, result from our study can be used as baseline for evaluation of vaccine implementation. In addition, due to the fact that rotavirus circulating similarity in Indonesia and those identified worldwide, it is likely that rotavirus vaccines available in the global market can be used to provide protection to young children in Indonesia.

## References

[CR1] Bishop RF, Unicomb LE, Soenarto Y, Suwardji H (1989). Ristanto, Barnes GL. Rotavirus serotypes causing acute diarrhoea in hospitalized children in Yogyakarta, Indonesia during 1978-1979. Arch Virol.

[CR2] Castello AA, Arvay ML, Glass RI, Gentsch J (2004). Rotavirus strain surveillance in Latin America: a review of the last nine years. Pediatr Infect Dis.

[CR3] Cuong NT, Minh NB, Anh DD, Thu NH, Tu NT, Nam TV, Thuy VT, Ogino M, Alam MM, Nakagomi T, Nakagomi O, Yamashiro T (2009). Molecular epidemiology of rotavirus diarrhoeae among children in Haipong, Vietnam: the emergence of G3 rotavirus. Vaccine.

[CR4] Das BK, Gentsch JR, Hoshino Y, Ishida S, Nakagomi O, Bhan MK, Kumar R, Glass RI (1994). Characterization of the G serotype and genogroup of New Delhi newborn rotavirus strain 116e. Virology.

[CR5] Esona MD, Steele D, Kerin T, Armah G, Peenze I, Geyer A, Page N, Nyangao J, Agbaya VA, Trabelsi A, Tsion B, Aminu M, Sebunya T, Dewar J, Glass R (2010). GentschJ. Determination of the G and P types of previously nontypeable rotavirus strains from the African Rotavirus Network, 1996–2004: identification of unusual G types. J Infect Dis.

[CR6] Esteban LE, Rota RP, Gentsch JR, Jiang B, Esona M, Glass RI, Glikmann G, Castello AA (2010). Molecular epidemiology of group A rotavirus in Buenos Aires, Argentina 2004–2007: reemergence of G2P[4] and emergence of G9P[8] strains. J Med Virol.

[CR7] Gentsch JR, Glass RI, Woods P, Gouvea V, Gorziglia M, Flores J, Das BK, Bhan MK (1992). Identification of group a rotavirus gene 4 types by Polymerase Chain Reaction. J Clin Microbiol.

[CR8] Gentsch JR, Laird AR, Bielfelt B, Griffin DD, Banyai K, Ramachandran M, Jain V, Cunliffe NA, Nakagomi O, Kirkwood CD, Fischer TK, Parashar UD, Bresee J, Jiang B, Glass RI (2005). Serotype diversity and reassortment between human and animal rotavirus strains: implications for rotavirus vaccine programs. J Infect Dis.

[CR9] Glass RI, Bresee JS, Turcios R, Fischer TK, Parashar UD, Steele AD (2005). Rotavirus vaccines: targeting the developing world. J Infect Dis.

[CR10] Glass RI, Parashar UD, Bresee JS, Turcios R, Fischer TK, Widdowson MA, Jiang B, Gentsch JR (2006). Rotavirus vaccines: current prospects and future challenges. Lancet.

[CR11] Gouvea V, Glass RI, Woods P, Taniguchi K, Clark HF, Forrester B, Fang ZY (1990). Polymerase Chain Reaction amplification and typing of rotavirus Nucleic Acid from Stool Specimens. J Clin Microbiol.

[CR12] Kawai K, O’Brien MA, Goveia MG, Mast TC, El Khoury AC (2012). Burden of Rotavirus gastroenteritis and distribution of Rotavirus strains in Asia: a systematic review. Vaccine.

[CR13] Khamrin P, Maneekarn N, Malasao R, Nguyen TA, Ishida S, Okitsu S, Ushijima H (2010). Genotypic linkages of VP4, VP6, VP7, NSP4, NSP5 genes of rotaviruses circulating among children with acute gastroenteritis in Thailand. Infect Genet Evol.

[CR14] Moe K, Thu HM, Oo WM, Aye KM, Shwe TT, Mar W, Kirkwood CD (2009). Genotyping of rotavirus isolates collected from children less than 5 years of age admitted for diarrhoea at the Yangon Children’s Hospital, Myanmar. Vaccine.

[CR15] Nyambat B, Meng CY, Vansith K, Vuthy U, Rin E, Kirkwood C, Bogdanovic-Sakran N, Kilgore PE (2009). Hospital-based surveillance for rotavirus diarrhoea in Phnom Penh, Cambodia, March 2005 trough February 2007. Vaccine.

[CR16] Ofoyo BA, Subekti D, Tjaniadi P, Machpud N, Komalarini S, Setiawan B, Simanjuntak C, Punjabi N, Corwin AL, Wasfy M, Campbell JR, Lesmana M (2002). Enteropathogens associated with acute diarrhea in community and hospital patients in Jakarta, Indonesia. FEMS Immunol Med Microbiol.

[CR17] Parashar UD, Hummelman EG, Bresee JS, Miller MA, Glass RI (2003). Global illness and deaths caused by rotavirus disease in children. Emerg Infect Dis.

[CR18] Parashar UD, Burton A, Lanata C, Boschi-Pinto C, Shibuya K, Steele D, Birmingham M, Glass RI (2009). Global Mortality Associated with Rotavirus Disease among Children in 2004. J Infect Dis.

[CR19] Putnam SD, Sedyaningsih ER, Listiyaningsih E, Pulungsih SP (2007). Komalarini, Soenarto Y, Salim O, Subekti D, Riddle MS, Burgess TH, Blair PJ. Group A Rotavirus-Associated Diarrhea in Children Seeking Treatment in Indonesia. J Clin Virol.

[CR20] Qazi R, Sultana S, Sundar S, Warraich H, un-Nisa T, Rais A, Zaidi AK (2009). Population-based surveillance for severe rotavirus gastroenteritis in children in Karachi, Pakistan. Vaccine.

[CR21] Radji M, Putnam SD, Malik A, Husrima R, Listyaningsih E (2010). Molecular characterization of human group A rotavirus from stool samples in young children with diarrhea in Indonesia. Southeast Asian J Trop Med Public Health.

[CR22] Santos N, Hoshino Y (2005). Global distribution of rotavirus serotypes/genotypes and its implication for the development and implementation of an effective rotavirus vaccine. Rev Med Virol.

[CR23] Simmond MK, Armah G, Asmah R, Banerjee I, Damanka S, Esona M, Gentsch JR, Gray JJ, Kirkwood C, Page N, Iturriza-Gomara M (2008). New oligonucleotide primers for P-typing of rotavirus strains: stategies for typing previously untypeable strains. J Clin Virol.

[CR24] Soenarto Y, Sebodo T, Ridho R, Alrasjid H, Rohde JE, Bugg HC, Barnes GL, Bishop RF (1981). Acute Diarrhea and Rotavirus Infection in Newborn Babies and Children in Yogyakarta, Indonesia, From June 1978 To June 1979. J Clin Microbiol.

[CR25] Soenarto Y, Aman AT, Bakri A, Waluya H, Firmansyah A, Kadim M, Martiza I, Prasetyo D, Mulyani NS, Widowati T (2009). Soetjiningsih, Karyana IP, Sukardi W, Bresee J, Widdowson MA. Burden of Severe Rotavirus Diarrhea in Indonesia. J Infect Dis.

[CR26] Wilopo SA, Kilgore P, Kosen S, Soenarto Y, Aminah S, Cahyono A, Ulfa M, Tholib A (2009). Economic evaluation of a routine rotavirus vaccination programme in Indonesia. Vaccine.

[CR27] World Health Organization (2002). Generic protocols for (i) hospital-based surveillance to estimate the burden of rotavirus gastroenteritis in children and (ii) a community-based survey on utilization of health care services for gastroenteritis in children.

